# Spontaneous splenic rupture as the initial symptom of splenic angiosarcoma: case report and literature review

**DOI:** 10.3389/fonc.2024.1366554

**Published:** 2024-05-02

**Authors:** Peiwu Jiang, Xiaowen Li, Jie Chen, Du Li, Yehong Han

**Affiliations:** ^1^ Department of Vascular Surgery, Hangzhou TCM Hospital Affiliated to Zhejiang Chinese Medical University, Hangzhou, China; ^2^ Department of General Surgery, Hangzhou TCM Hospital Affiliated to Zhejiang Chinese Medical University, Hangzhou, China; ^3^ Department of Pathology, Hangzhou TCM Hospital Affiliated to Zhejiang Chinese Medical University, Hangzhou, China

**Keywords:** angiosarcoma, immunohistochemistry, spleen, splenectomy, splenic rupture, case reprot

## Abstract

Spontaneous splenic rupture is an extremely rare occurrence, often attributed to tumorous pathologies. Among these, primary splenic angiosarcoma stands as a malignancy arising from the endothelial cells within the spleen. While sporadic cases have been reported globally, there remains a lack of comprehensive consensus on standardized approaches for diagnosis and treatment. We report a case of an 83-year-old male who underwent emergency enhanced CT due to sudden shock, revealing significant intra-abdominal fluid accumulation. Emergency surgery revealed splenic rupture necessitating splenectomy. Histopathological examination confirmed the diagnosis of splenic angiosarcoma. Despite successful surgery, the patient succumbed to severe complications two weeks postoperatively.

## Introduction

The spleen, a crucial immune organ, rarely presents with tumors, whether they’re primary or secondary malignancies. Primary angiosarcoma of the spleen, also known as hemangiosarcoma, was initially documented by Langhans in 1879 (1). Reported cases of primary splenic angiosarcoma in English literature are fewer than 300. Despite its high malignancy, there’s a scarcity of comprehensive guidance in diagnosis and treatment, with only isolated experiences shared. In this context, we detail a case where the diagnosis was confirmed through splenectomy following spontaneous splenic rupture and subsequent hemorrhage. This case report aims to contribute insights into the diagnostic and therapeutic trajectory of this condition. It is noteworthy that the bleeding event occurred three days after initiating anticoagulant therapy.

## Case description

An 83-year-old elderly male presented with abdominal discomfort. He has a history of hypertension and atrial fibrillation, managed with long-term oral amlodipine, maintaining good blood pressure control. However, he was not receiving anticoagulant therapy. He has no other chronic underlying diseases or family history of genetic disorders. Physical examination revealed no significant abnormalities. Investigations showed a significant elevation in D-dimer levels (16.85 mg/L) and a decrease in platelet count (37 * 10^9/L). Ultrasonography revealed splenomegaly with diffuse changes of undetermined etiology. CTPA suggested suspicious embolism in the pulmonary artery branches. Despite the bleeding risk, a cautious decision was made to initiate low-dose low molecular weight heparin anticoagulant therapy after weighing the risks and benefits. Due to concerns regarding excessive contrast agent use, an immediate enhanced CT scan of the spleen was not performed.

**Figure 1:**
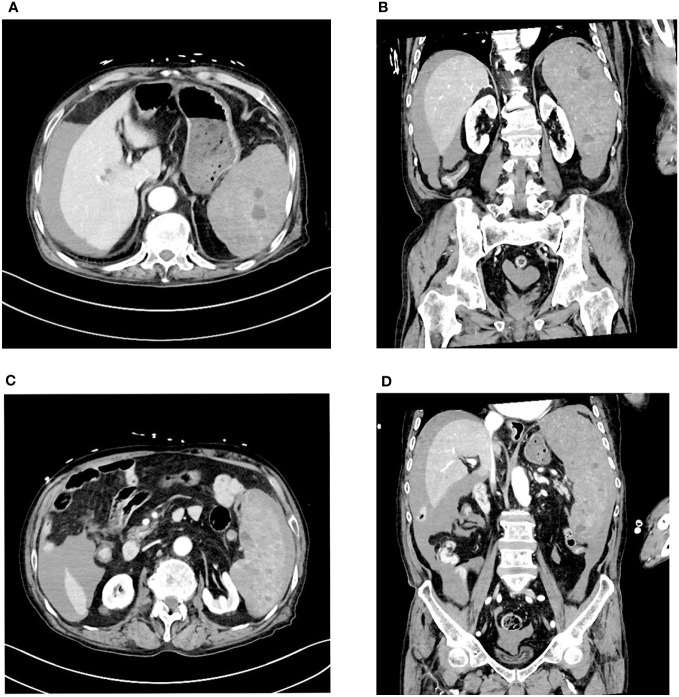
Enhanced CT scan reveals a large amount of fluid accumulation in the abdominal cavity. Multiple low-density nodules are visible within the spleen, accompanied by uneven density below the spleen. **(A, B)** Multiple low-density nodules are visible within the spleen. **(C, D)** Uneven density changes are observed on the inner side of the spleen.

**Figure 2:**
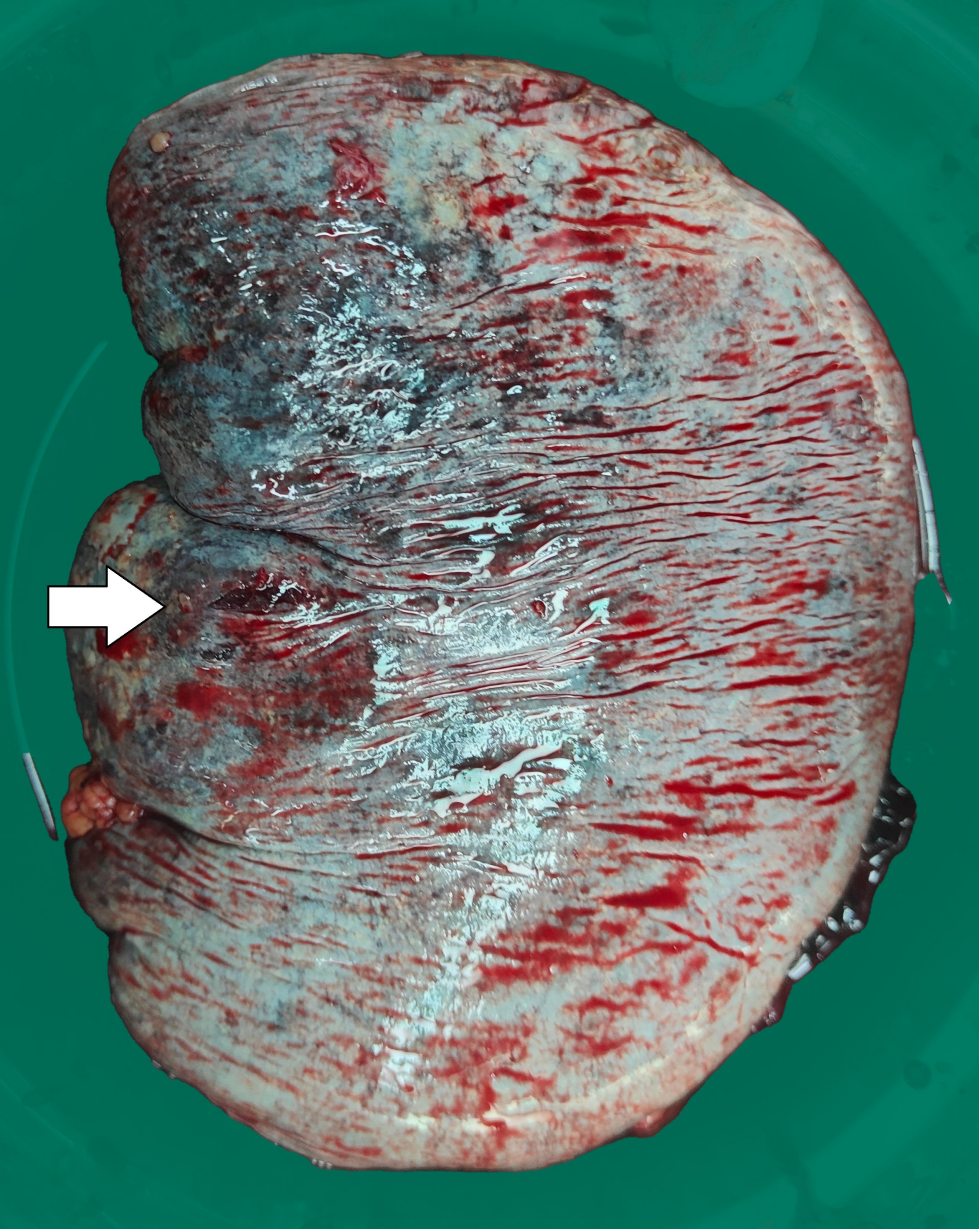
The surgically removed spleen shows an approximately 0.5cm rupture with uneven surface coloration.

Three days later, the patient suddenly experienced severe left-sided abdominal pain accompanied by hypotension and tachycardia. Despite receiving large volumes of fluid resuscitation over several hours, there was no improvement. Emergency enhanced CT scan revealed a significant intra-abdominal hemorrhage with splenomegaly and multiple low-density lesions within the splenic parenchyma, showing mild enhancement on contrast administration. Initial consideration was given to intra-abdominal visceral bleeding (**Figure 1**). Emergency exploratory laparotomy unveiled around 4000ml of intraperitoneal hemorrhage and an actively bleeding splenic rupture (**Figure 2**), necessitating splenectomy. The patient received significant transfusions of packed red blood cells and plasma during the surgery. The surgery was timely and successful, rescuing the patient from hemorrhagic shock.

Following postoperative care, the patient was admitted to the intensive care unit (ICU) for hemodynamic stabilization, antimicrobial therapy, and adjunctive interventions. Subsequently, the patient underwent implantation of an inferior vena cava (IVC) filter for lower extremity deep vein thrombosis and underwent gastric repair surgery for stress ulcer perforation. Despite these measures, the patient remained hemodynamically unstable with persistent lactate accumulation. Regrettably, further therapeutic interventions were declined by the patient’s family, and the patient succumbed to his condition two weeks postoperatively.

**Figure 3:**
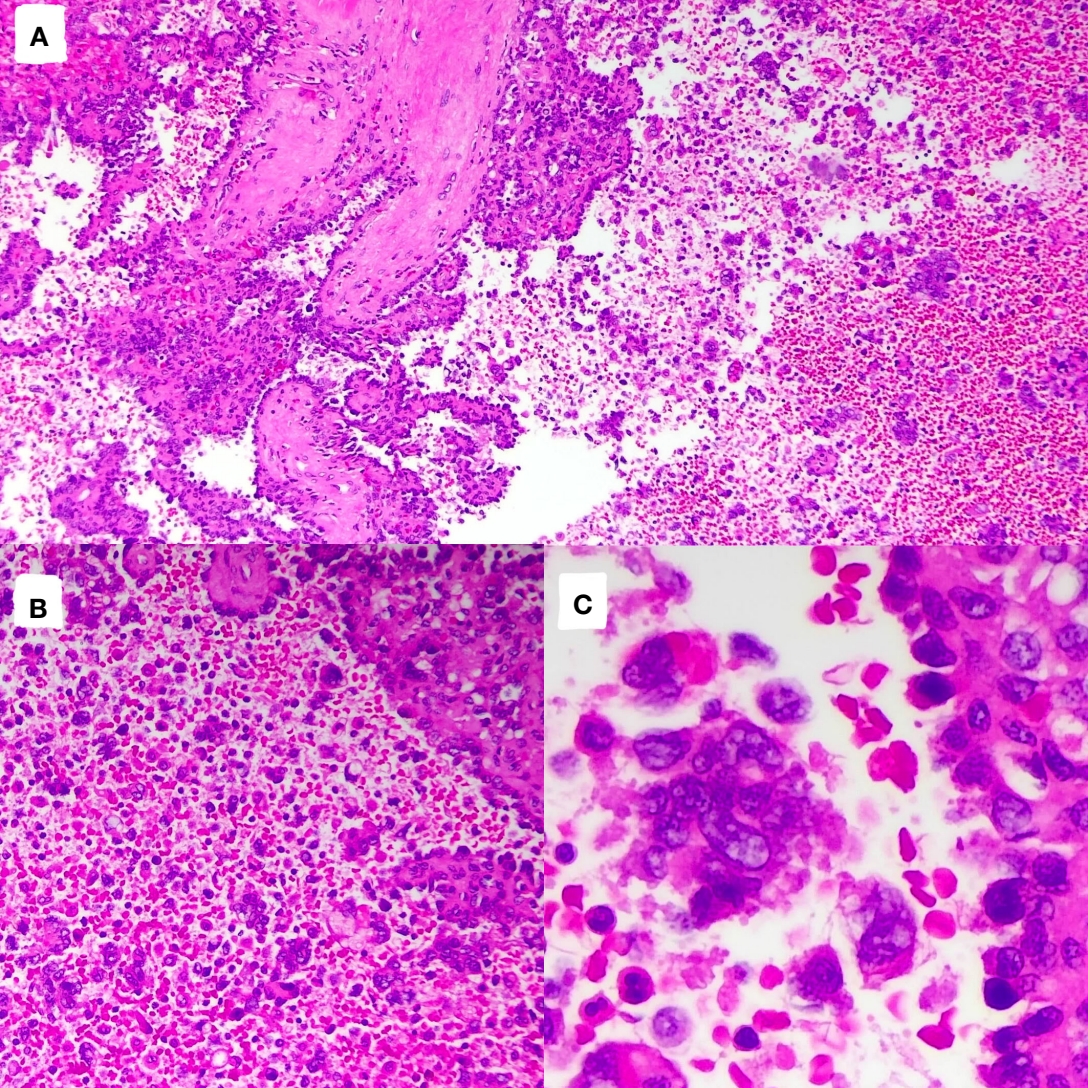
Pathological images and immunohistochemistry staining were performed, indicating significant tumor cell heterogeneity with visible nuclear division. [**(A)**, HE×100]; [**(B)**, HE×200]; [**(C)**, HE×400].

**Figure 4:**
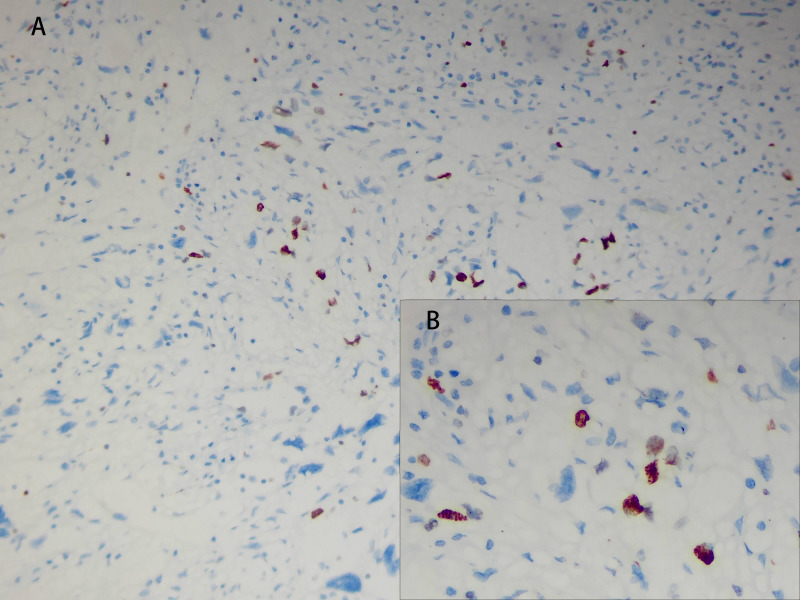
The Ki-67 positive index is approximately 10-15%. [**(A)**, ×A00]; [**(B)**, HE×400].

The tumor cells in the spleen display significant heterogeneity and visible mitotic figures. Considering the Hematoxylin and Eosin (HE) staining (**Figure 3**) and immunohistochemical markers, the primary consideration is a vascular sarcoma. Immunohistochemical staining revealed partial positivity for CD31, CD8, ERG, FLI-1, Vimentin, with a Ki-67 index of 10-15% (**Figure 4**). Conversely, the tumor tested negative for CD68, D2-40, CD123, CD138, CD56, CD4, CD8, CD20, CD21, CD3, CD30, CD5, and BCL-6.

## Discussion

Splenic angiosarcoma originates from endothelial cells within blood vessels and exhibits significant invasiveness (2). Consequently, it is associated with high rates of local recurrence, systemic metastasis, and a short overall survival duration. Common sites of metastasis include intrasplenic spread and dissemination to the liver, lungs, and lymph nodes. There have also been reports of metastasis to the bone marrow, causing symptoms resembling hematological disorders. In certain cases, leukocytosis and thrombocytosis may occur (3).

According to statistical data, 80% of patients survive only six months following spontaneous splenic rupture, indicating a poor treatment outcome (4). Patients who undergo splenectomy before rupture have a slightly better prognosis, with survival extending up to 11 months (5). The increased risk of immediate mortality might be due to hypovolemic shock and disseminated intravascular coagulation caused by the rupture (6). It also heightens the risks of peritoneal and hematogenous dissemination. Montemayor et al. found that patients with splenic angiosarcoma who underwent splenectomy before rupture, rather than after rupture, had longer survival times (14.4 months vs. 4.4 months) (7).

The accurate preoperative diagnosis of this condition presents considerable challenges. Employing biopsy carries inherent risks, including the potential for bleeding and malignant dissemination (6). Consequently, histological confirmation typically occurs post-splenectomy, a procedure serving both diagnostic and therapeutic purposes.

Clinically, primary splenic angiosarcoma lacks distinctive symptoms, often presenting as anemia, splenomegaly accompanied by abdominal discomfort (75%), fatigue, weight loss, fever, hepatomegaly, splenomegaly, anemia, and thrombocytopenia. Approximately 30% of cases manifest with spontaneous splenic rupture as the initial symptom (8). There is an emerging perspective indicating that thrombocytopenia might serve as the primary symptom, even in the absence of anomalies in other laboratory tests.

While a conclusive diagnosis might not be achievable, the majority of angiosarcoma patients exhibit indications on imaging studies. The prevalent findings on ultrasound or CT scans encompass splenomegaly and inconclusive solid alterations within the spleen (9). Computed tomography illustrates splenomegaly accompanied by single or multiple nodular masses, displaying heterogeneous low attenuation. Lesion margins might manifest irregular or indistinct features, with contrast-enhanced scans revealing heterogeneous enhancement within the tumor.

Laboratory investigations linked to this condition commonly include anemia, thrombocytopenia, leukocytosis, and elevated erythrocyte sedimentation rate (1, 10). Apart from anemia, our patients do not present with these anomalies. Tumor markers (AFP, CEA, CA-125, and CA19-9) consistently remain within normal ranges or exhibit only slight elevations.

While challenging and without the potential for complete cure, surgical intervention remains the foremost therapeutic approach for splenic angiosarcoma. Determining the appropriate indications for surgically managing primary splenic tumors and splenic metastases presents inherent difficulties. Primary angiosarcoma or undifferentiated pleomorphic sarcoma, given their highly malignant nature, necessitate urgent surgical intervention. In cases where spleen involvement is limited in metastatic disease, splenectomy is considered a component of palliative measures when symptoms are prominent or as part of cytoreductive surgery (11).

Chemotherapy (such as taxanes, doxorubicin, liposomal doxorubicin, and ifosfamide) has been the primary treatment modality for advanced stage angiosarcoma. Anthracyclines like doxorubicin, doxorubicin analogs, and paclitaxel serve as active monotherapy options (12, 13). Reports on radiotherapy are scarce; however, based on observations from treating angiosarcomas in other locations, radiotherapy’s efficacy tends to be inferior compared to other types of sarcomas.

In our scenario, the patient initially reported discomfort in the upper abdomen. Despite multiple examinations showing nodular changes in the spleen, their exact nature remained uncertain. Given the concurrent diagnosis of pulmonary embolism, an immediate splenectomy wasn’t possible. Emergency surgery was eventually conducted after severe hemorrhagic shock, significantly impacting the patient’s overall survival. Hence, caution is necessary when encountering an enlarged spleen alongside low platelet count. When circumstances permit, opting for a preventive splenectomy appears to be a more cautious approach.

## Conclusion

In our case, the presenting symptomatology comprised epigastric discomfort. Despite undergoing several examinations revealing nodular alterations within the spleen, conclusive determination of their etiology remained elusive. Given the concurrent diagnosis of pulmonary embolism, prompt planning for splenectomy was impeded.

Despite the emergent surgery performed for massive hemorrhagic shock, it undoubtedly precipitated a severe deterioration in survival time. Given the markedly elevated D-dimer levels and the diagnosis of pulmonary embolism, anticoagulant therapy was instituted. Subsequent to this intervention, there was an amelioration in D-dimer levels compared to baseline, albeit this may have precipitated the onset of the major bleeding event. Therefore, splenomegaly coupled with thrombocytopenia should raise suspicion, and radiographic abnormalities of the spleen should alert to the possibility of splenic neoplasms. Even in the presence of indications for anticoagulation, the use of anticoagulants should be approached cautiously.

## Data availability statement

The original contributions presented in the study are included in the article/supplementary material. Further inquiries can be directed to the corresponding author.

## Ethics statement

The studies involving humans were approved by Ethics Committee of Hangzhou Hospital of Traditional Chinese Medicine. The studies were conducted in accordance with the local legislation and institutional requirements. The participants provided their written informed consent to participate in this study. Written informed consent was obtained from the individual(s) for the publication of any potentially identifiable images or data included in this article. Written informed consent was obtained from the participant/patient(s) for the publication of this case report.

## Author contributions

PJ: Formal analysis, Writing – original draft, Writing – review & editing, Data curation, Investigation. XL: Writing – review & editing, Funding acquisition. JC: Writing – original draft. DL: Visualization, Writing – original draft. YH: Conceptualization, Supervision, Writing – original draft, Writing – review & editing.
